# Greedy parameter optimization for diabatic quantum annealing

**DOI:** 10.1098/rsta.2021.0416

**Published:** 2023-01-23

**Authors:** Tadashi Kadowaki, Hidetoshi Nishimori

**Affiliations:** ^1^ DENSO CORPORATION, Kounan, Minato-ku, Tokyo 108-0075, Japan; ^2^ Institute of Innovative Research, Tokyo Institute of Technology, Yokohama, Kanagawa 226-8503, Japan; ^3^ Graduate School of Information Sciences, Tohoku University, Sendai, Miyagi 980-8579, Japan; ^4^ RIKEN Interdisciplinary Theoretical and Mathematical Sciences Program (iTHEMS), Wako, Saitama 351-0198, Japan

**Keywords:** quantum annealing, counterdiabatic driving, greedy optimization

## Abstract

A shorter processing time is desirable for quantum computation to minimize the effects of noise. We propose a simple procedure to variationally determine a set of parameters in the transverse-field Ising model for quantum annealing (QA) appended with a field along the y-axis. The method consists of greedy optimization of the signs of coefficients of the y-field term based on the outputs of short annealing processes. We test the idea in the ferromagnetic system with all-to-all couplings and spin-glass problems, and find that the method outperforms the traditional form of QA and simulated annealing in terms of the success probability and the time to solution, in particular, in the case of shorter annealing times, achieving the goal of improved performance while avoiding noise. The non-stoquastic $\sigma^{y}$ term can be eliminated by a rotation in the spin space, resulting in a non-trivial diabatic control of the coefficients in the stoquastic transverse-field Ising model, which may be feasible for experimental realization.

This article is part of the theme issue ‘Quantum annealing and computation: challenges and perspectives’.

## 1. Introduction

Quantum optimization algorithm is an active field of research, including quantum annealing (QA) [1–8], adiabatic quantum computing [9] and quantum approximate optimization algorithm (QAOA) [10]. If QA operates in the adiabatic regime, it often encounters a difficulty of exponential computation time coming from exponential closing of the energy gap due to a first-order phase transition, examples of which are found in [11–13]. A number of approaches have been tried to mitigate this problem, including non-stoquastic Hamiltonians [14–17], inhomogeneous field driving [18–23], reverse annealing and pausing [24–34] and counterdiabatic driving [35–49].

The last method of counterdiabatic driving is particularly appealing because it allows us to reduce the computation time when compared with the adiabatic process, yet keeping the success probability large. The reduction of computation time is important not just theoretically but also experimentally from the viewpoint of avoiding the effects of noise in quantum devices. One of the drawbacks of the idea of counterdiabatic driving is the excessive complexity of the additional counterdiabatic term, see e.g. [40], which generally makes it impossible to be implemented experimentally. An interesting development to avoid this problem is the variational approach to optimize the coefficients of approximate counterdiabatic terms composed of manageable operators [44–46,49–51]. In the present paper, we further simplify this strategy and propose to determine just the signs of coefficients of counterdiabatic terms composed of y components of the Pauli matrix. Since the determination of signs is processed in a greedy way variationally, we call the method quantum greedy optimization (QGO). This approach is appealing because of its simplicity and yet a significantly improved success probability in comparison with the original QA and the classical method of simulated annealing (SA).

This paper is organized as follows. Section 2 defines the model system and explains our strategy to solve optimization problems. The performance of the method is analysed numerically in §3 for the mean-field, ferromagnetic and spin glass problems, and the results are compared with SA [52] and the original QA. Also discussed are a further simplification of the algorithm and a possible improvement based on an oracle setting. The final §4 summarizes and discusses the results.

## 2. Formulation

Let us define the following time-dependent Hamiltonian:

2.1 $$H=A(t)H^{z}+B(t)H^{x}+\sum_{i=1}^{N}C_{i}(t)H_{i}^{y},$$

where $H^{z}$, $H^{x}$ and $H_{i}^{y}$ are the problem (Ising) Hamiltonian, the transverse field term (to be called the x-field), and the y-field term, respectively,

2.2 $$H^{z}=-\sum_{i<j}J_{ij}\sigma_{i}^{z}\sigma_{j}^{z},\;H^{x}=-\sum_{i=1}^{N}\sigma_{i}^{x}\;\mathrm{and}\;H_{i}^{y}=-\sigma_{i}^{y}.$$

The symbols $\sigma_{i}^{x},\sigma_{i}^{y}$ and $\sigma_{i}^{z}$ denote the components of the Pauli matrix at site (qubit) i, and $J_{ij}$ is for the interaction between sites i and j. The problem size is N. The term $H_{i}^{y}$ renders the system non-stoquastic [53] and was introduced in [45] to approximately realize counterdiabatic driving. See also [44,48,50,54]. The time-dependent coefficients A(t),B(t) and $C_{i}(t)$ should satisfy the initial (t=0) and final (t=τ) conditions,

2.3 $$A(0)=C_{i}(0)=0,\;B(0)\neq 0,\;A(\tau )>0\;\mathrm{and}\;B(\tau )=C_{i}(\tau )=0$$

such that the system starts with the simple transverse field term $H^{x}$ and ends with the problem Hamiltonian $H^{z}$. The y-field is applied only in the middle of annealing. We arbitrarily choose the following functions for these coefficients for simplicity though other (similar) functions can be considered [45],

2.4 $$A(t)=\frac{at}{\tau },\;B(t)=b(1-\frac{t}{\tau })\;\mathrm{and}\;C_{i}(t)=c_{i}\sin^{2}(\frac{\pi t}{\tau })$$

with a=1 to set the overall energy scale. The annealing time τ can be chosen arbitrarily and we test the cases of τ=1 and τ=5 in the following.

Our strategy is to optimize b and $c_{i}$ under a variational principle to optimize appropriate measures. We simplify the process by fixing the amplitude of $c_{i}$ to a value found optimal in the mean-field case (to be described below) and choosing only the sign of $c_{i}$ in a greedy way. This facilitates the process considerably and yet will turn out to lead to significant performance improvements.

Our variational optimization is carried out by minimization of the expectation value of the final energy $E=\left\langle \psi (t=\tau )|H^{z}|\psi (t=\tau )\right\rangle$, where ψ(t) is the wave function at time t, as well as by maximization of the ground state probability, or the fidelity, $P_{\mathrm{gs}}=|\left\langle \psi (t=\tau )|\psi_{\mathrm{gs}}\right\rangle {|}^{2}$, where $|\psi_{\mathrm{gs}}\rangle$ is the true ground state of the Ising Hamiltonian. Since we do not know the true ground state in a generic problem, we refer to the latter measure as an oracle.

The additional y-field term may not be straightforward to be implemented experimentally. Nevertheless, by a simple rotation of the spin axes [45,50],

2.5 $$U_{g}(t)=\exp\left(\frac{i}{2}\sum_{i}\theta_{i}(t)\sigma_{i}^{z}\right)$$

with $\theta_{i}(t)=\arctan(C_{i}(t)/B(t))$, we can transform the Hamiltonian into a transverse-field Ising model with non-trivial time dependence of coefficients,

2.6 $$H_{\mathrm{eff}}=A(t)H^{z}-\frac{1}{2}\sum_{i}\frac{d\theta_{i}(t)}{dt}\sigma_{i}^{z}-\sum_{i}\sqrt{B{\left(t\right)}^{2}+C_{i}{\left(t\right)}^{2}}\sigma_{i}^{x},$$

which may be easier to realize experimentally than the original Hamiltonian of equation (2.1). An example of the behaviour of the coefficients in equation (2.6) is shown in appendix A.

## 3. Numerical results

In this section, we present numerical results for the mean-field, ferromagnetic and random (spin glass) systems. In addition, we discuss an additional algorithm which shortcuts the process of iterative determination of the signs of coefficients.

### (a) Mean-field theory

As a preliminary to the next section of a ferromagnetic system, we first analyse the properties of the mean-field theory with the Hamiltonian (see [44] for a related idea)

3.1 $$H=-t\left\langle \sigma^{z}\right\rangle \sigma^{z}-b(1-t)\sigma^{x}-c\sin^{2}(\pi t)\sigma^{y},$$

where $\left\langle \sigma^{z}\right\rangle$ is the magnetization of the system $\left\langle \psi (t)|\sigma^{z}|\psi (t)\right\rangle$. We set τ=1 in this section. The state vector can be expressed as a qubit |ψ(t)⟩=α|0⟩+β|1⟩, where $|0\rangle ={\left(1,0\right)}^{T}$ and $|1\rangle ={\left(0,1\right)}^{T}$, and the Schrödinger equation reads

3.2 $$\frac{d}{dt}\left(\begin{array}{c} \alpha \\ \beta \end{array}\right)=\left(\begin{array}{cc} it\left\langle \sigma^{z}\right\rangle & ib(1-t)+c\sin^{2}(\pi t)\\ ib(1-t)-c\sin^{2}(\pi t) & -it\left\langle \sigma^{z}\right\rangle \end{array}\right)\left(\begin{array}{c} \alpha \\ \beta \end{array}\right),$$

where $\left\langle \sigma^{z}\right\rangle$ is $|\alpha {|}^{2}-|\beta {|}^{2}$. As the ground state of the final Hamiltonian is doubly degenerate, we choose the spin-up state $\left\langle \sigma^{z}\right\rangle >0$ as the desired ground state. Two measures of success as defined in the previous section, the fidelity $P_{\mathrm{gs}}=(\left\langle \sigma^{z}\right\rangle +1)/2$ and the energy $E=-{\left\langle \sigma^{z}\right\rangle }^{2}$, can be expressed by the magnetization $\left\langle \sigma^{z}\right\rangle$ in the mean-field theory, which is not the case generally.

**Figure 1.**
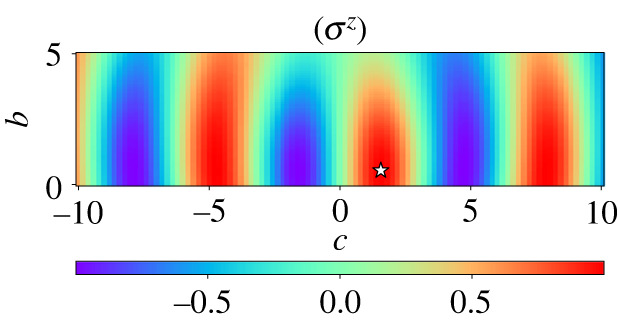
Parameter dependence of the magnetization $\left\langle \sigma^{z}\right\rangle$ for the mean-field theory. The optimal point $\left(b_{\mathrm{opt}}^{\mathrm{mf}}=0.539,c_{\mathrm{opt}}^{\mathrm{mf}}=1.565\right)$ is marked in an open asterisk. (Online version in colour.)

Figure 1 shows the dependence of magnetization $\left\langle \sigma^{z}(t=1)\right\rangle$ on b and c. In this landscape of the parameter space, we find the optimal values to maximize $\left\langle \sigma^{z}\right\rangle$ by the classical optimizer, the Broyden–Fletcher–Goldfarb–Shanno (BFGS) algorithm [55–58], as $b_{\mathrm{opt}}^{\mathrm{mf}}=0.539$ and $c_{\mathrm{opt}}^{\mathrm{mf}}=1.565$ with $\left\langle \sigma^{z}\right\rangle =1.000$. Notice that no greedy search is applied here since the problem is very simple in the mean-field case. Although finding optimal values of the coefficients is sufficient for our purpose in the following analysis, we illustrate in appendix B the time developments of the wave function, the coefficients, and the magnetization in the mean-field context, comparing mean-field results with the exact solution of the counterdiabatic term, for better understanding of the physics of the mean-field theory.

### (b) Ferromagnetic system

#### (i) Parameter dependence of the energy and fidelity

We move a step forward and analyse the ferromagnetic system with all-to-all interactions,

3.3 $$H^{z}=-\frac{J}{N-1}\sum_{i<j}\sigma_{i}^{z}\sigma_{j}^{z}.$$

The mean-field theory of the previous section is expected to give the correct solution to this model in the thermodynamic limit [59] though finite-size effects may reveal differences.

**Figure 2.**
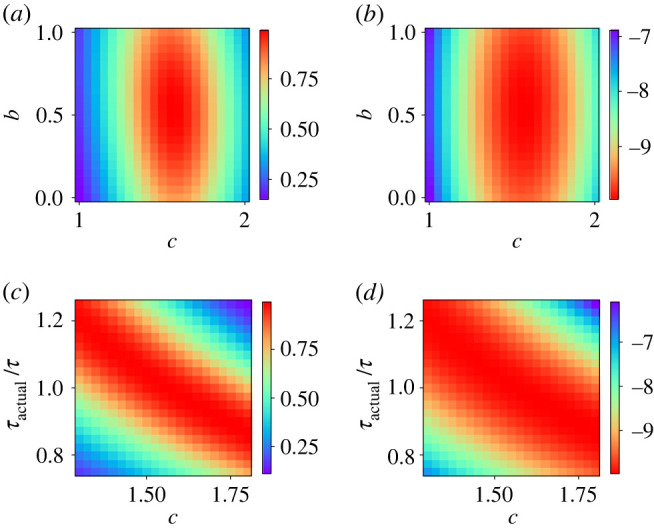
Parameter (b,c) dependence of (*a*) the fidelity and (*b*) the energy in the ferromagnetic model with N=20. Dependence on the annealing time and the parameter c of (*c*) the fidelity and (*d*) the energy when annealing is terminated before the designated annealing time ($\tau_{\mathrm{actual}}<\tau$) and is carried through beyond ($\tau_{\mathrm{actual}}>\tau$). Colour scales are located on the right side of each figure. (Online version in colour.)

As the interactions are homogeneous, we drop the i dependence of $c_{i}$ and set all of them to c. To break the $Z_{2}$ symmetry, we choose positive values of b and c and focus our attention on the region around the optimal value obtained in the mean-field case, b∈[0,1] and c∈[1,2]. Dependence of the fidelity and energy on b and c is shown in figure 2*a*,*b*, respectively, for N=20. We also conducted the same analysis for the case where annealing is terminated before the designated annealing time ($\tau_{\mathrm{actual}}<\tau$) or carried through beyond ($\tau_{\mathrm{actual}}>\tau$), and the results are displayed in figure 2*c*,*d*. These plots indicate that the optimization is robust in b and sensitive to c and the actual annealing time. Similarities of figure 2*a*,*b* and of figure 2*c*,*d* suggest that the two measures of success, the fidelity and the energy, give essentially the same result, as anticipated from the mean-field case.

**Figure 3.**
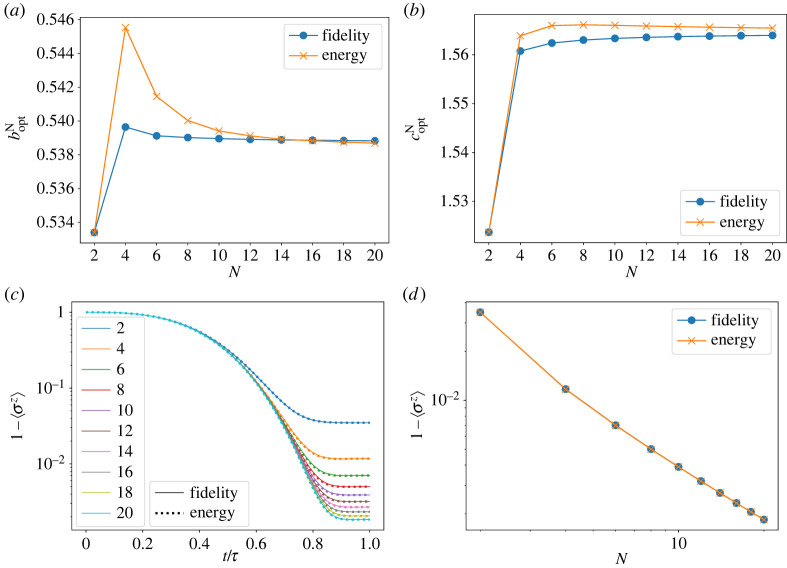
(*a*) Size dependence of the parameter $b_{\mathrm{opt}}^{N}$ optimized with respect to the fidelity and energy measures in the ferromagnetic model, (*b*) size dependence of the optimized parameter $c_{\mathrm{opt}}^{N}$, (*c*) time development of the magnetization and (*d*) size dependence of the magnetization. (Online version in colour.)

System size dependence of the optimal parameter values for each N, $b_{\mathrm{opt}}^{N}$ and $c_{\mathrm{opt}}^{N}$, is shown in figure 3*a*,*b*. They show convergence beyond N≈12 towards the values at N=20 ($b_{\mathrm{opt}}^{N=20}=0.539,c_{\mathrm{opt}}^{N=20}=1.564$). These values are in good agreement with the results from the mean-field theory, $b_{\mathrm{opt}}^{\mathrm{mf}}=0.539$ and $c_{\mathrm{opt}}^{\mathrm{mf}}=1.565$, as expected. The error in magnetization $1-\left\langle \sigma^{z}\right\rangle$ decreases with the system size, and we do not see a visible difference between the two measures in figure 3*c*,*d*. The residual error decreases polynomially as a function of the system size N. We will use the size-dependent parameters $b_{\mathrm{opt}}^{N}$ and $c_{\mathrm{opt}}^{N}$ obtained from fidelity optimization for further analysis.

#### (ii) Greedy optimization of the parameters

In preparation of the random case, we next try a step-wise greedy optimization of parameters assuming site dependence of the sign of $c_{i}$ as in the well-established methods like quadratic pseudo-Boolean optimization and the roof duality algorithm solutions [60–63] and a sampling-based algorithm [64]. More precisely, we choose the optimal values of $c_{i}$ one by one with other coefficients fixed at their constant values. This approach reduces the search space considerably and also makes the energy (or fidelity) landscape simple.

**Figure 4.**
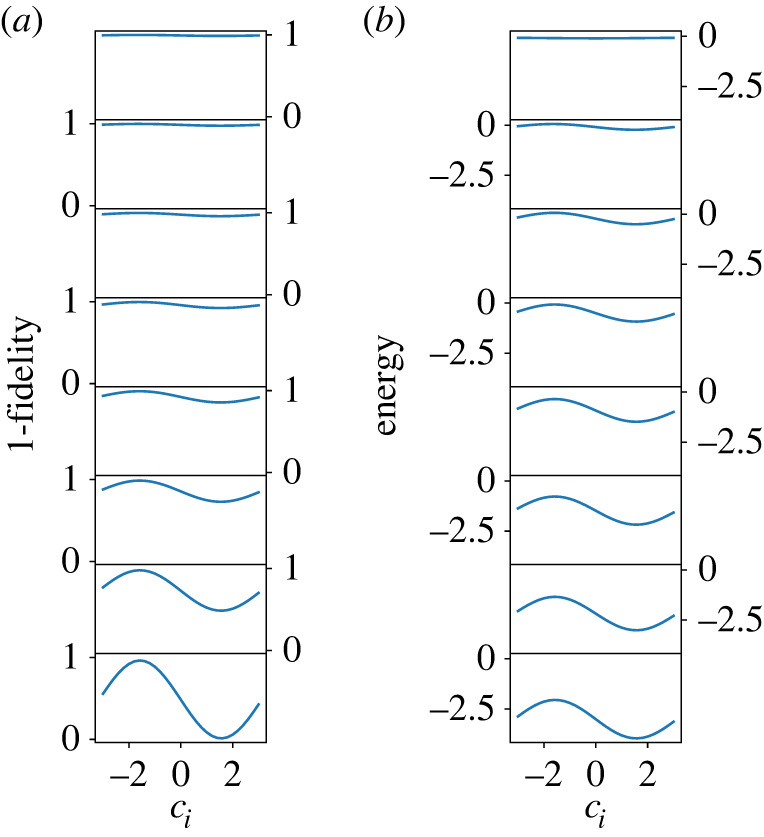
The parameter $c_{i}$ dependence of (*a*) the fidelity and (*b*) the energy in each iteration (from top to bottom) for the ferromagnetic model with N=8. Note that the site index i changes from step to step as explained in the text. See also figure 14 in which the same data are shown but with different vertical scales for different iteration steps for better resolution at each step. (Online version in colour.)

The first step is to pick up an arbitrary i (e.g. i=1) and optimize $c_{1}$ by setting other $c_{j}(j\neq 1)$ to zero. As shown in the rows of figure 4*a*,*b* for N=8, we choose optimal $c_{1}$ by minimizing $1-P_{\mathrm{gs}}$ (in (*a*)) or E (in (*b*)). See also figure 14, where the scale of the vertical axis is changed step by step for a better resolution. In practice, when we use E as the function to be minimized, this function is symmetric with respect to the change of sign of $c_{1}$ due to the double degeneracy of states. We therefore arbitrarily set $c_{1}$ to a positive value $c_{1}^{\mathrm{BFGS}}$ by BFGS. When we use $1-P_{\mathrm{gs}}$ for minimization, we choose the all-up state as the target ground state, so no problem of degeneracy exists. We next choose another i (e.g. i=2) and optimize $c_{2}$ by fixing $c_{1}$ to the already-optimized value and other $c_{j}\;(j\neq 1,2)$ to zero. The $c_{2}$ dependence of $1-P_{\mathrm{gs}}$ and E is shown in the second row of figure 4. This process is repeated until all parameters are fixed. It is seen in figure 4 that $1-P_{\mathrm{gs}}$ and E become smaller as the iteration proceeds.

We have found that the optimal values $c_{i}^{\mathrm{BFGS}}$ are always close to $c_{\mathrm{opt}}^{N}$: The resulting optimal values vary from site to site, ranging from 1.507 to 1.566, while $c_{\mathrm{opt}}^{N=8}$ is 1.563 according to the analysis of the previous section. This means that our sequential greedy optimization strategy finds the ground state with good precision for the ferromagnetic system with all-to-all couplings.

### (c) Spin glass problem

We apply essentially the same procedure as developed above to the prototypical hard optimization problem of spin glass.

The algorithm proceeds as follows. The value of b is set to $b_{\mathrm{opt}}^{N}$ obtained for the ferromagnetic case, and the absolute value of $c_{i}$ is fixed to $c_{\mathrm{opt}}^{N}$ as demonstrated in the ferromagnetic case. We only choose the sign of each $c_{i}$ step by step, site to site, in a greedy way. It will be seen that this simple process leads to significant improvements in performance as compared to the traditional QA and classical SA.

The sign of $c_{i}$ is chosen by the average gradient of the optimization measure (E or $1-P_{\mathrm{gs}}$) near the origin $0\leq c_{i}\leq \Delta =0.1$ with the values of other $c_{j}$’s (j≠i) fixed. The reason is that the optimization measure has only a single minimum, whose position can be detected near the origin, as a function of a single $c_{i}$, as will be illustrated later.

More precisely, we first choose an arbitrary i (e.g. i=8) and fix all other $c_{j}$ to zero. The optimization measure E is symmetric with respect to the inversion $c_{8}\to -c_{8}$, and we therefore look instead at the curvature of E in the asymmetric range $0\leq c_{8}\leq \Delta$. This process is repeated for all i and we choose the site i with the largest absolute average curvature. If this turns out to be i=8 with a positive derivative, we set $c_{8}=-c_{\mathrm{opt}}^{N}$, which breaks the symmetry. We next choose another i (e.g. i=6) and see the sign of the average derivative of E near the origin with $c_{8}$ fixed to $-c_{\mathrm{opt}}^{N}$ and all other $c_{i}$’s to zero. We choose another i and the same process is repeated for all i(=1,2,3,4,5,7) and we select the site with the largest value of the absolute average derivative (e.g. i=6 with a negative derivative) and assign the sign as $c_{6}=+c_{\mathrm{opt}}^{N}$. Iterating this process, all $c_{i}$’s are assigned fixed values. See algorithm 1.


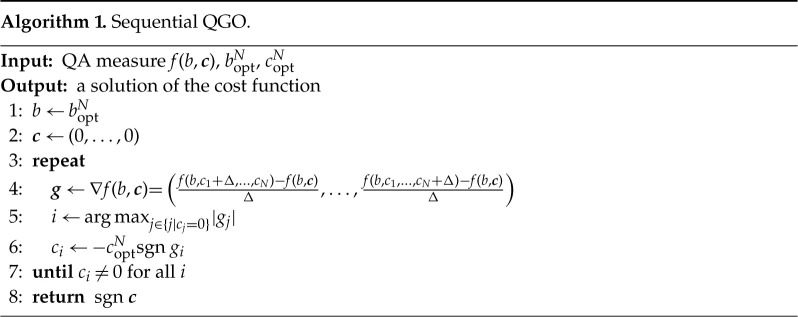


**Figure 5.**
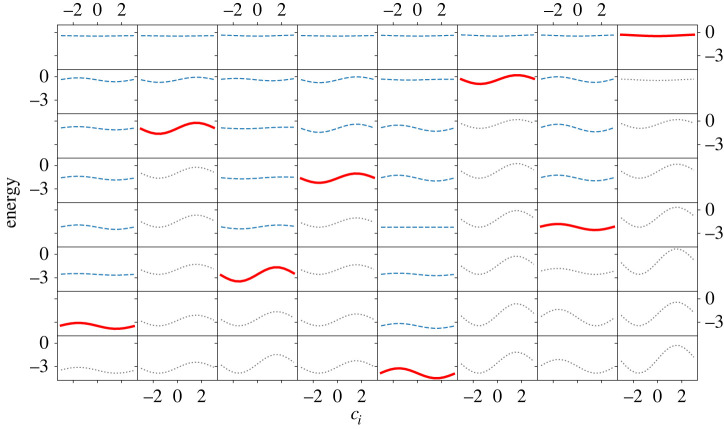
Parameter dependence of the energy measure in each iteration of sequential QGO in the random model (N=8) from the first step (top row) to the last step (bottom row). Each column represents a site i, 1 (leftmost column) to 8 (rightmost column). Blue-dashed and red-bold curves are parameters for sites that are under evaluation in each iteration. In each iteration, the site with the largest absolute average gradient marked in red bold is selected to fix the parameter. Grey-dotted curves located under the red curves are additional plots for the site which we do not evaluate for further iterations. See also figure 15 in which the same data are shown with different vertical scales for different iteration steps for a better resolution. (Online version in colour.)

An example of this process is illustrated in figure 5 for the all-to-all coupling Sherrington–Kirkpatrick model of spin glass [65] with the distribution function of interaction,

3.4 $$P(J_{ij})∼\frac{1}{\sqrt{2\pi \sigma^{2}}}\exp\left(-\frac{J_{ij}^{2}}{2\sigma^{2}}\right),$$

with $\sigma^{2}=1/(N-1)$. Red curves show E as a function of a given $c_{i}$ which has the largest absolute value of the average gradient. Note that the procedure works for the top row for the initial step as the average gradients are calculated in the asymmetric range [0,Δ] and resulted in all positive even though the curve is symmetric at $c_{i}=0$. Blue dotted curves are for other sites with smaller gradient, and light grey curves show the behaviour of E when the already-fixed $c_{i}$ is tentatively changed with other $c_{i}$’s fixed. One sees that the minimum value of E on red curves decreases as the greedy optimization proceeds from the top row to the bottom row.

The final values of $c_{i}$ thus obtained are compared with those from direct brute-force optimization by the BFGS algorithm,

$$c_{\mathrm{QGO}}=(1.563,-1.563,-1.563,-1.563,1.563,-1.563,1.563,-1.563)$$

and

$$c_{\mathrm{BFGS}}=(1.570,-1.564,-1.562,-1.567,1.569,-1.561,1.561,-1.563).$$

We observe that the difference is minimal, at most 0.4%, and the signs are correctly reproduced by the greedy algorithm.

**Figure 6.**
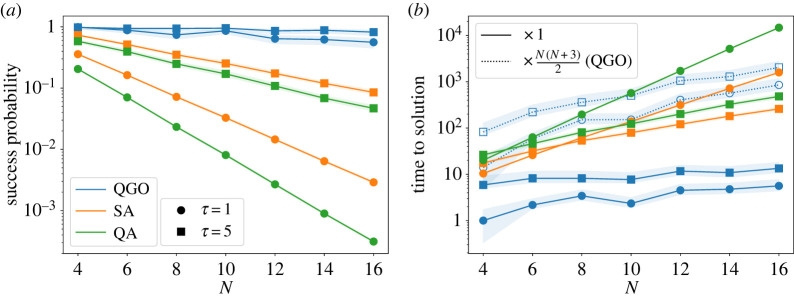
(*a*) Size dependence of the average success probability of QGO (blue), SA (orange), and QA (green) for τ=1 (circle) and 5 (square) in 100 instances of the random system. (*b*) Size dependence of time to solution of QGO (blue), SA (orange) and QA (green) for τ=1 (circle) and 5 (square). Dotted lines with open markers are plotted by taking into account the factor N(N+3)/2 multiplying the original QGO to compensate for multiple runs of QA. The shaded region represents 95% confidence intervals for each line. (Online version in colour.)

For a more systematic analysis, we applied the algorithm to the spin glass with various sizes from N=4 to 16 with 100 random instances for each N. The resulting success probability is plotted as a function of size in figure 6*a* for τ=1 and 5. QGO and QA obey the Schrödinger dynamics, and the calculation was conducted by QuTiP [66]. Also plotted are the success probabilities by the traditional QA and SA. The latter classical algorithm has been tested by the following schedule of temperature decrease (inverse temperature increase)

3.5 $$\beta (t)=\frac{\left(t/\tau \right)}{1.1-(t/\tau )},$$

where β(0)=0 and β(τ)=10 and by solving the classical master equation. QGO returns not a state vector in QA or a probability distribution in SA but a specific solution of spin configuration, which is the output from the algorithm 1. Thus the success probability of QGO refers to a portion of instances with success. Confidence intervals are calculated by bootstrapping from 10 000 resamples. It is clear that the present algorithm QGO far outperforms the other two.

Another viewpoint is provided by the time-to-solution (TTS), a standard measure of computation time for heuristic algorithms [67]. It is defined as

3.6 $$\mathrm{TTS}=\tau \frac{\log(1-P)}{\log(1-p_{\mathrm{success}})},$$

where $p_{\mathrm{success}}$ is the empirical probability of success for the computation time τ, and P is the target success probability often set to 0.99. The result is plotted in figure 6*b*, which apparently shows a low computational cost of the present algorithm QGO drawn in blue. We should note, however, that QGO carries an overhead of repeated computation of the parameter dependence of E at each iteration.

QGO calls QA as a subroutine N(N+3)/2 times in total to determine the gradients. This number is derived as follows: in the n-th iteration (n=1,…,N), we run QA at $\left(c_{1},\ldots ,c_{N}\right)$ once and at $\left(c_{1},\ldots ,c_{i}+\Delta ,\ldots ,c_{N}\right)$ for ith site N−(n−1) times as we already fixed n−1 sites. Thus, the total number is $\sum_{n=1}^{N}(1+N-n+1)=N(N+3)/2$. After we take into account this overhead, the TTS of QGO becomes comparable with the other two as seen in figure 6*b* in blank blue symbols. It is nevertheless concluded that QGO achieves much better success probability with similar computation times.

**Figure 7.**
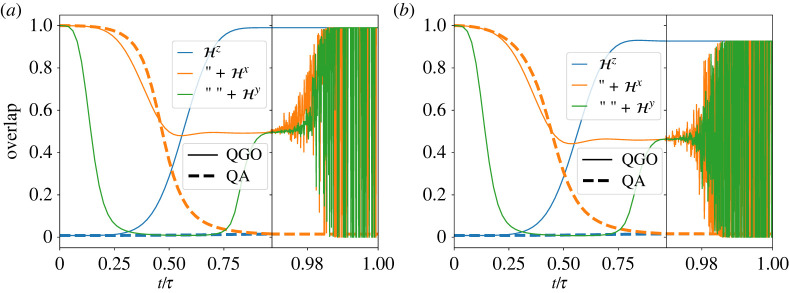
Time dependence of overlaps of the wave function with the instantaneous ground states of three kinds of the Hamiltonian, (blue) the final Ising model $H^{z}$, (orange) the transverse-field Ising model $A(s)H^{z}+B(s)H^{x}$ and (green) the full Hamiltonian $A(s)H^{z}+B(s)H^{x}+C(s)H^{y}$, for (solid) QGO and (dashed) QA. The problems are (*a*) the ferromagnetic system, and (*b*) a spin glass problem, both with eight spins. Curves in the range [0.97, 100] beyond the black vertical line are magnified for better resolution. (Online version in colour.)

In order to further understand how the algorithm leads to better results, we have analysed the time dependence of overlaps of the wave function with the instantaneous ground states of three kinds of the Hamiltonian, (i) the final Ising model $H^{z}$, (ii) the transverse-field Ising model $A(s)H^{z}+B(s)H^{x}$, and (iii) the full Hamiltonian $A(s)H^{z}+B(s)H^{x}+C(s)H^{y}$, for QGO and QA. Results are plotted in figure 7*a* for the ferromagnetic system and (*b*) for a spin glass problem. It is observed that QGO does not directly let the system follow the instantaneous ground state of the full Hamiltonian but it succeeds in enhancing the overlap with the final Ising Hamiltonian at a relatively early stage of computation.

### (d) Further simplifications of the algorithm

To see if we can further simplify the algorithm without much compromising the performance, we test two possibilities in this section.

#### (i) y-Field optimization

**Figure 8.**
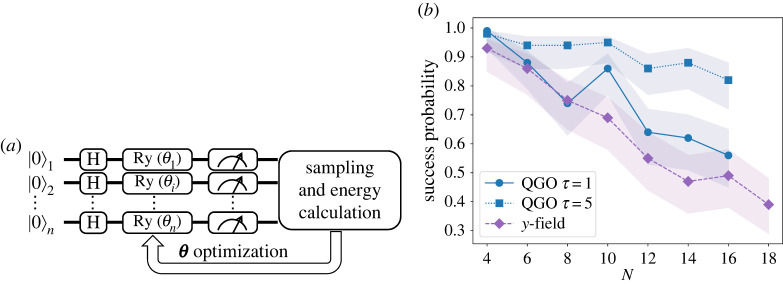
(*a*) Quantum circuit of the y-field optimization. (*b*) Size dependence of the success probability of QGO (blue) for τ=1 (circle) and 5 (square) and the y-field optimization (purple diamond). The shaded region represents 95% confidence intervals for each line. (Online version in colour.)

The first example is to drive the state only by $H^{y}$, i.e. by a rotation around the y-axis,

3.7 $$U(\theta )=\exp\left(\frac{i}{2}\sum_{i}\theta_{i}(t)\sigma_{i}^{y}\right)\equiv \prod_{i}R_{y}(\theta_{i})$$

starting from the ground state of $H^{x}$ and to variationally optimize the parameters θ to minimize E as illustrated symbolically in figure 8*a*. Results for 100 spin glass instances are shown in figure 8*b*. It is seen that this y-field optimization performs slightly worse than QGO for τ=1. It is also noted by comparison with figure 6*a* that the y-field optimization leads to better results than QA and SA with τ=1. Thus the present method may be useful for some purposes due to its simplicity and its easiness of implementation possibly on a gate-based hardware.

#### (ii) Single-shot QGO

Let us next study what happens if we fix the signs of coefficients of $c_{i}$ in a single shot without iteration. We first fix one of the parameters, e.g. $c_{1}=c_{\mathrm{opt}}^{N}$ (spin-up), to break the $Z_{2}$ symmetry. Signs of other sites are next fixed according to the gradient of the optimization function, $1-P_{\mathrm{gs}}$ or E, near the origin as shown in algorithm 2.


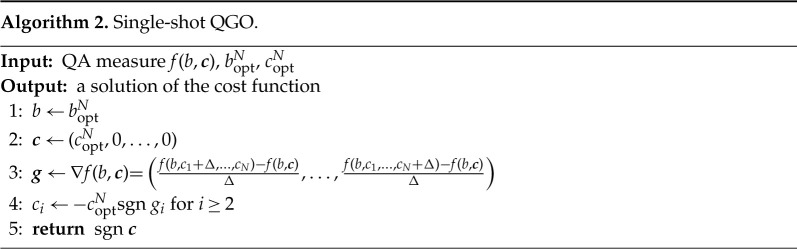


**Figure 9.**
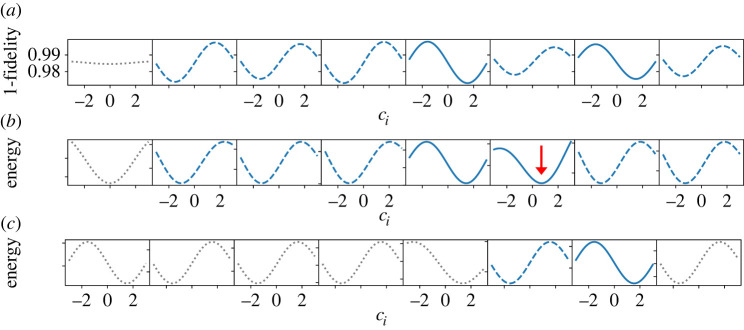
$c_{i}$ dependence of the single-shot QGO with (*a*) $1-P_{\mathrm{gs}}$ and (*b*) E for optimization in an eight-spin glass problem. (*c*) The sixth and seventh parameters are re-evaluated by fixing other parameters to $\pm c_{\mathrm{opt}}$ according to the results in (*b*). Blue-dashed and solid curves represent that the optimal values are negative and positive, respectively. Grey-dotted curves indicate re-evaluation of the already fixed parameters similarly to figure 5. Vertical scales are normalized for each estimation in (*b*,*c*). (Online version in colour.)

Figure 9*a* shows the $c_{i}$ dependence of $1-P_{\mathrm{gs}}$ for each spin to confirm our assumption that the optimal $c_{i}$ is close to $\pm c_{\mathrm{opt}}^{N}$. Blue-dashed and solid curves represent that the optimal values are negative and positive, respectively. Grey-dotted curves indicate re-evaluation of the fixed-parameter, similarly to figure 5. The correct solution has been obtained by this single-shot QGO using $1-P_{\mathrm{gs}}$ for this instance. When the energy measure E is employed for optimization as shown in figure 9*b*, two spins (the sixth and seventh) are fixed incorrectly. As illustrated in figure 9*c*, the ground state is retrieved if the two spins are re-evaluated after the other six spins are fixed. This suggests that we need iterations of the process for better results if we use E for optimization.

It is also noted that minimum values in the sixth spin in figure 9*b* are not around $\pm c_{\mathrm{opt}}^{N=8}=\pm 1.563$ but are around 0.7 (red arrow). This implies that our assumption, optimal values are around $\pm c_{\mathrm{opt}}^{N}$ is not valid in this case, which would lead to the difference in the performance between (*a*) and (*b*).

**Figure 10.**
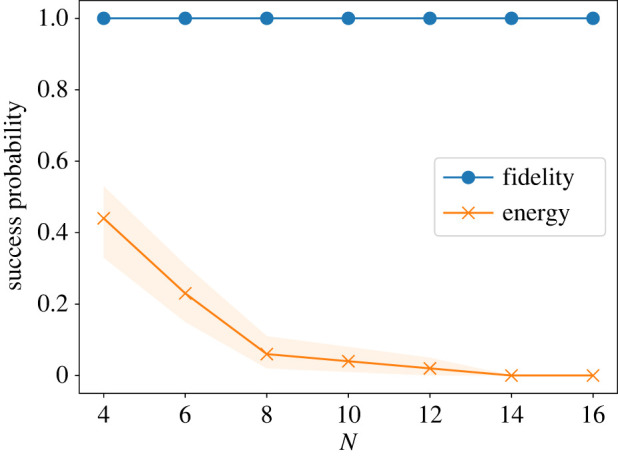
Size dependence of the success probability of the single-shot QGO with fidelity (blue circle) and energy (orange x) measures averaged out the 100 instances of spin glass problem. The shaded region represents 95% confidence intervals for each line. (Online version in colour.)

**Figure 14.**
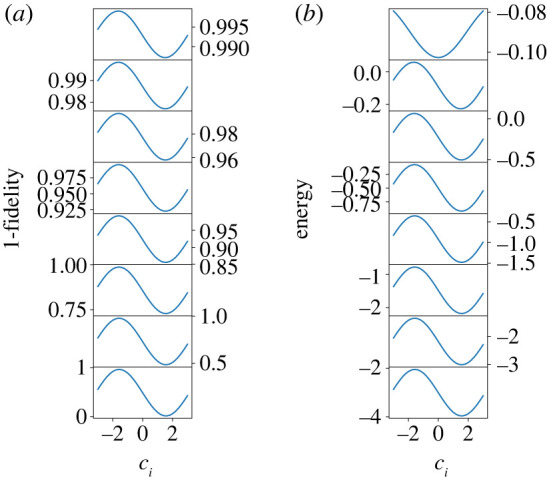
The same plot as in figure 4 with different vertical scales. (Online version in colour.)

Figure 10 shows the performances of the single-shot QGO for two measures of optimization for 100 spin glass instances. The single-shot QGO with the fidelity measure always succeeds in identifying the ground state, while the success probability quickly dumps under the energy measure. The fidelity measure is the overlap between the final state and the ground state and thus includes information of the ground state, leading to better results.

## 4. Summary and discussion

We have formulated a variational algorithm of QGO aiming to achieve improved performance of QA through a simple process of adjustment of signs of y-field coefficients. The result showed notable improvements in the success probability over the original method as well as over classical SA under comparable computational costs. Since we adjust the signs of coefficients sequentially, the energy landscape of each step is very simple with only a single minimum at almost the same absolute values of coefficients, the adjustment or optimization process does not encounter the problems of barren plateau in deep variational quantum circuits [68] or highly complicated landscapes which plague well-known variational algorithms such as VQE and QAOA [69]. Another advantage of the present method is the simplicity of the additional term in the Hamiltonian, the y-field, which can be rewritten in terms of the transverse-field Ising model without *y*-field by a rotation in the spin space. The latter can be implemented experimentally if the hardware can be designed to allow for the non-monotonic time dependence of coefficients of the x field and the Ising part of the Hamiltonian. It is in principle possible to apply the same idea to a final Hamiltonian with x- and y-components of the Pauli matrix, typically for problems of quantum state preparation in chemistry, although it is non-trivial if the present protocol would lead to satisfactory results in such cases. In the real settings of quantum processors, we cannot measure the fidelity because the target state is unknown. The fidelity analysis provides the algorithm’s upper limit, revealing that the success probability can be 100% and suggesting that the goodness of the cost function defines the algorithm’s performance. This finding is informative for the further development of algorithms.

## Figures and Tables

**Figure 11.**
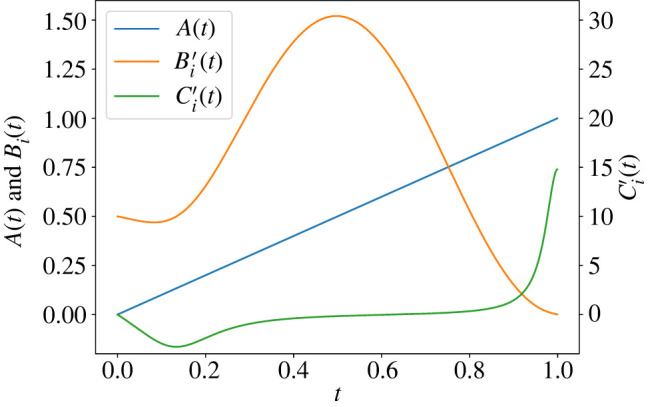
Time development of the coefficients A(t), $B_{i}^{\prime }(t)$ and $C_{i}^{\prime }(t)$ for a=1, b=0.5, $c_{i}=1.5$ and τ=1. (Online version in colour.)

**Figure 12.**
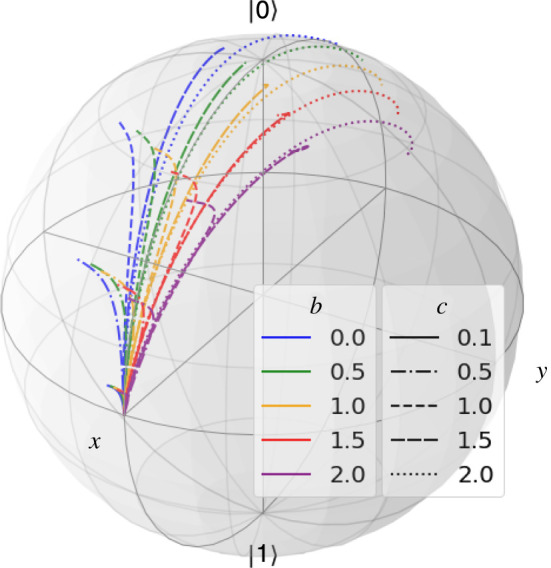
Trajectories of the system under the mean-field theory for b= (0.0, 0.5, 1.0, 1.5, 2.0) and c= (0.1, 0.5, 1.0, 1.5, 2.0). (Online version in colour.)

**Figure 13.**
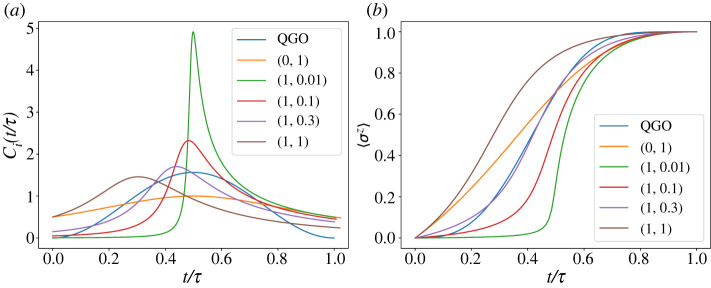
Exact solution of (*a*) counterdiabatic Y-field, and (*b*) magnetization for QGO and (g,h)=(0,1), (1,0.01), (1,0.1), (1,0.3) and (1,1). (Online version in colour.)

**Figure 15.**
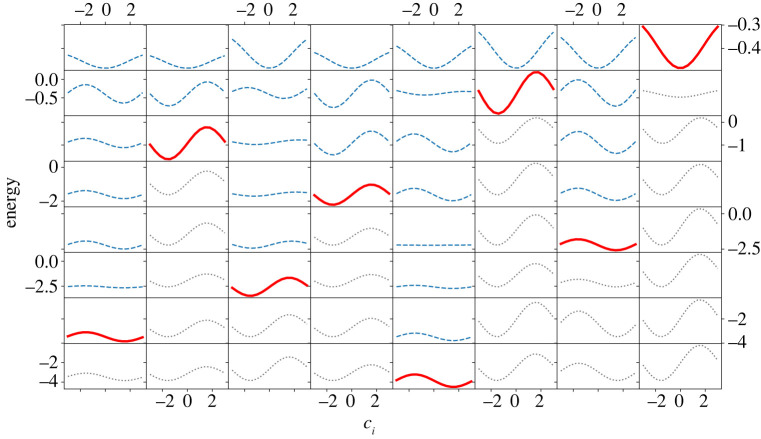
Same plot as in figure 5 with different scales of vertical axis. (Online version in colour.)

## Data Availability

The code and data are provided in electronic supplementary material [70].

## Appendix A. Time dependence of the coefficients in the rotated frame

An example of the behaviour of the coefficients in the rotated frame, equation (2.6), is given in this appendix. Let us write the Hamiltonian as

A 1 $$H_{\mathrm{eff}}=A(t)H^{z}-\sum_{i}B_{i}^{\prime }(t)\sigma_{i}^{x}+\sum_{i}C_{i}^{\prime }(t)\sigma_{i}^{z},$$

where

A 2 $$B_{i}^{\prime }(t)=\sqrt{B{\left(t\right)}^{2}+C_{i}{\left(t\right)}^{2}}$$

and

A 3 $$C_{i}^{\prime }(t)=-\frac{bc_{i}[\pi (1-t/\tau )\sin(2\pi t/\tau )+\sin^{2}(\pi t/\tau )]}{2\tau [b^{2}{\left(1-t/\tau \right)}^{2}+c_{i}^{2}\sin^{4}(\pi t/\tau )]}.$$

Figure 11 shows the time dependence of A(t), $B_{i}^{\prime }(t)$ and $C_{i}^{\prime }(t)$ for a=1, b=0.5, $c_{i}=1.5$ and τ=1. It is observed that $B^{\prime }(t)$ and $C^{\prime }(t)$ are non-monotonic, which suppresses system’s transition to excited states in the final state.

## Appendix B. System behaviour of the mean-field theory

To visualize the system behaviour under the mean-field theory discussed in §3a, we plot the trajectory of the system on the Bloch sphere in figure 12 for a number of parameter values with b=0.5 (green) and c=1.5 (long dashed) being closest to the optima. The system starts along the x-axis and is supposed to end ideally at the north pole |0⟩. The optimal parameter values are observed to approximately follow a straightforward path connecting these points.

Figure 13 compares the exact counterdiabatic driving function for the mean-field theory [40] and our approximate result. The parametrized Ising Hamiltonian is

B 1 $$H^{z}=-g\left\langle \sigma^{z}\right\rangle \sigma^{z}-h\sigma^{z}.$$

Although QGO tested in the main text is g=1 and h=0, the parameter h should be non-zero in the exact calculation to avoid the double degeneracy of states. Exact solutions are obtained for (g,h)= (0,1), (1,0.01), (1,0.1), (1,0.3) and (1,1). The system follows the instantaneous ground state under these exact solutions whereas it does not under our approximate scheme, to which (g,h)=(1,0.3) shows the closest behaviour.

## Appendix C. Enhanced scales of performance measure

For better resolution of the vertical axes of figures 4 and 5, we show in figures 14 and 15 the same data with enhanced scales of vertical axes.

## Appendix D. y-Field optimization

The simple y-field optimization algorithm can be executed the circuit model in figure 8*a*, and described as follows:
